# Evaluating the Tumor Burden, Histological Changes, and Immune Landscape of Breast Cancer Post-neoadjuvant Chemotherapy: Insights From 50 Cases

**DOI:** 10.7759/cureus.80258

**Published:** 2025-03-08

**Authors:** Arasi Rajesh, Dharma Saranya Gurusamy, Rajalakshmi Manickam

**Affiliations:** 1 Pathology, Government Medical College, Tuticorin, IND; 2 Pathology, Government Medical College, Virudhunagar, IND

**Keywords:** breast cancer, immune response, post-neoadjuvant chemotherapy, proliferation index, residual cancer burden, tumor-infiltrating lymphocytes, tumor regression

## Abstract

Breast cancer is a heterogeneous disease with variable responses to neoadjuvant chemotherapy (NACT). Evaluating the histopathological and immune changes in post-NACT breast cancer specimens is crucial for understanding treatment response and guiding further management. This study aims to assess tumor burden using the Residual Cancer Burden (RCB) index, examine histological alterations, evaluate immune activity through tumor-infiltrating lymphocytes (TILs), and analyze proliferative capacity via Ki-67 expression in post-NACT breast cancer specimens.

A cross-sectional study of 50 modified radical mastectomy (MRM) specimens post-NACT was conducted. The histopathological analysis included tumor regression changes, stromal and cellular alterations, and nodal involvement. Immune response was assessed by quantifying TILs, and proliferation was measured using the Ki-67 index. Statistical correlations were made between clinicopathological parameters, TILs, and Ki-67 expression.

Residual disease was detected in 39 cases (78%), and 11 cases (22%) had no residual disease. Among the 39 cases with residual disease, the majority were classified as RCB II (22 cases, 56%), 16 cases (41%) were classified as RCB III, and one case (3%) was classified as RCB I. Common histological changes post-NACT included fibrosis in 31 cases (62%), necrosis in 19 cases (38%), and infiltration by foamy histiocytes in 16 cases (32%). Malignant epithelial cells more frequently exhibited foamy cytoplasm (16 cases (41%) vs. two cases (5%); p=0.0003), hyperchromatic nucleus (26 cases (67%) vs. six cases (15%); p=0.0001), and prominent nucleoli (26 cases (67%) vs. four cases (10%); p=0.0001) compared to benign cells. Among the 39 cases with residual disease, low TIL and high Ki-67 expression were observed in 20 cases (51%), while 12 cases (32%) showed high TIL and low Ki-67. Residual tumors with high TIL and high Ki-67 (four cases, 10%) and low TIL and low Ki-67 (three cases, 8%) were less common. A significant inverse relationship was found between TIL levels and Ki-67 expression (p=0.0002), as tumors with low TIL were more likely to have high Ki-67 expression (20 cases, 51%), whereas those with high TIL more frequently exhibited low Ki-67 expression (12 cases, 32%).

Post-NACT evaluation of tumor burden, immune landscape, and proliferation provides valuable prognostic insights. Integrating RCB, TILs, and Ki-67 into routine pathological assessment may aid patient stratification and guide personalized treatment strategies. Further large-scale studies are needed to validate these findings and improve therapeutic decision-making in breast cancer management.

## Introduction

Breast cancer is a complex disease with diverse histological and molecular characteristics. Globally, it is the second most common cancer, constituting 11.6% of all cancers and 6.9% of cancer deaths [1]. In India, the most common cancer among women is breast cancer, with an overall prevalence of 77.9 per 100,000 population and a mortality rate of 10.7% [2]. The advent of molecular profiling techniques has enabled the identification of distinct intrinsic subtypes with varying prognoses and therapeutic responses. Neoadjuvant chemotherapy (NACT) has the potential to downstage tumors and enable breast conservation surgery and is the current modality of treatment adopted for early and locally advanced breast cancers [3,4]. The histopathological reporting of post-NACT specimens is challenging due to therapy-induced changes in breast parenchyma, which can mimic atypical lesions, obscure residual disease, and complicate the assessment of tumor margins and nodal involvement [4]. The impact of neoadjuvant therapy on the histological and immunological landscapes of residual disease remains incompletely understood. Pathological complete response (pCR) is a robust predictor of favorable prognosis in breast cancer patients. Breast cancer is molecularly heterogeneous, and the response rate to NACT is heterogeneous across different breast cancer subtypes. In cases of residual disease, the Residual Cancer Burden (RCB) index provides valuable prognostic information, correlating with overall survival (OS) and event-free survival (EFS) [5].

Modified radical mastectomy (MRM) specimens post-NACT also provide an opportunity for the in vivo assessment of tumor response and identification of factors to refine the risk stratification of patients. The progression of the residual tumor post-chemotherapy depends on the proliferative capacity of the residual tumor cells, their interaction with the tumor microenvironment, and the chemotherapy-induced immunomodulation of the tumor immune landscape. Thus, assessing tumor-infiltrating lymphocytes (TILs) [6,7] and proliferative capacity [8] as biomarkers to stratify patients and identify patients for additional systemic treatments may be a valid and relevant option. Patients with high lymphocytic infiltrates in residual disease experienced significantly increased metastasis-free five-year survival and OS than patients with lesser TIL [9]. Increased Ki-67 values in residual tumors in non-responders to NACT are known to be a poor prognostic factor [10]. This study aims to evaluate the histopathological changes in tumor and stromal tissues following NACT, estimate the tumor burden using the RCB index, assess immune activity via TIL quantification, and determine proliferative capacity through Ki-67 expression. By correlating these parameters with clinicopathological factors, we seek to provide insights into the prognostic implications of NACT in breast cancer management. Our study tries to establish the feasibility of comprehensive evaluation of residual disease in post-NACT specimens. The findings may shed light on the biological mechanisms underlying treatment response and resistance and inform the development of more individualized therapeutic strategies.

## Materials and methods

This cross-sectional study was done in the Department of Pathology in a tertiary care center, Government Medical College Hospital Tirunelveli, Tamil Nadu, India. Formalin-fixed specimens and paraffin-embedded tissue blocks of breast carcinoma patients who underwent radical surgery as per standard operating protocol post-NACT were included in the study. The study included 50 cases diagnosed between 2019 and 2020. Written and informed consent was obtained from the patients enrolled in the study. Clinical data of the patients, including age, laterality, results of imaging studies including ultrasound, magnetic resonance imaging (MRI), preoperative biopsy findings, and treatment details, were collected from the archived patient records. The Tirunelveli Government Medical College Institutional Ethics Committee (TIREC) approved the study with reference number 1266/PATHO/2018.

Histopathological analysis

Gross and microscopic evaluation of the tumor bed was done as per protocol [11,12]. The specimens were sliced at 5 mm intervals. In samples with grossly discernible tumors, the maximum tumor dimension was measured in two orthogonal diameters. Distance from the margins was measured by inking the margins. In specimens with extensive fibrotic and hyalinized areas, bits were taken, and the sampled foci were marked with colored pins. The maximum tumor dimensions in these cases were measured, correlating with the microscopic findings. Lymph nodes were isolated, and the maximum dimensions were measured. Microscopic examination was done for the assessment of pathological response, histological changes, cancer cellularity, and the presence of ductal carcinoma in situ (DCIS). The RCB method [11] was used to assess pathological response. Maximum tumor size, cancer cellularity, percentage of in situ disease, number of nodes, and diameter of the largest metastasis were used to calculate the RCB. Stromal changes evaluated were fibrosis, infiltration by hemosiderin-laden macrophages, foamy histiocytic infiltrates, necrosis, angiogenesis, hyalinization of blood vessels, calcification, and cholesterol clefts. The residual malignant tumor cells and the benign epithelial cells were evaluated for the foamy appearance of cytoplasm, cytoplasmic shrinkage, increased nuclear-cytoplasmic ratio, hyperchromasia, and prominent nucleoli. Histological changes in lymph nodes evaluated were residual disease, fibrosis, vascular proliferation, and histiocytic infiltration. The percentage of TIL was assessed by evaluating the percentage of stromal areas occupied by mononuclear cells in the boundaries of residual tumor cells, adopting the guidelines recommended by the International Immuno-Oncology Biomarker Working Group on Breast Cancer [7]. The hematoxylin and eosin (H&E) sections were scanned at 50-100×, and the TIL percentage was estimated to be a continuous variable at 200-400×. In cases with multiple foci of residual disease, the average TIL cellularity from the different microscopic fields was calculated. Cases were defined as high TIL when the TIL percentage was >60% and low when the percentage was <60%, validated by previous protocols [9]. Tissue sections (8 μm) were incubated for five minutes in 3% hydrogen peroxide to quench endogenous tissue peroxidase. Antigen retrieval was performed in citrate buffer (pH 6), using an electric pressure cooker set at 120° C for five minutes. Primary mouse monoclonal antibodies were directed against Ki-67 (mouse monoclonal MIB-1; DAKO) in 1:1000 dilution. The tissue sections were incubated with primary antibodies for 25 minutes at room temperature. The unbound primary antibodies were washed and treated with commercial biotinylated secondary anti-immunoglobulin, followed by avidin coupled with biotinylated horseradish peroxidase. Immunohistochemical reactions were visualized using chromogen diaminobenzidine (DAB). Sections were counterstained with hematoxylin after immunohistochemistry. Tissue sections with defined expressions and absence of expressions were used as positive and negative controls, respectively. Three foci of maximum Ki-67 labeling fields were identified in low power. The maximum Ki-67 labeling areas were examined in high power (400×), and the proliferation index was calculated as the percentage of 100 cells counting at least 1000 cells. Based on the Ki-67 levels, the residual disease was classified into low (0-15%), intermediate (15.1-35%), and high (35.1-100%) posttreatment Ki-67 levels as per previous studies [13]. All the slides were evaluated by two independent observers who were blinded to the clinicopathological data. In cases of deviations, the observers analyzed the sections together and arrived at a consensus.

Statistical analysis

Descriptive analysis was performed, and the data were presented as numbers (percentages) for categorical variables and means (standard deviations) for continuous variables. The stromal TIL percentage was stratified into two categories: TIL high and TIL low. The Ki-67 labeling index was stratified into low, intermediate, and high. The statistical significance of the associations of the clinicopathological features and TIL grades and Ki-67 was compared using the chi-squared test and Fisher's exact test for categorical variables and one-way ANOVA for continuous variables, with a p-value of <0.05 as the significance threshold. All the statistical tests were performed via IBM SPSS Statistics for Windows, Version 29.0.2.0 (Released 2023; IBM Corp., Armonk, New York, United States).

## Results

Clinicopathological features

Table 1 highlights the clinicopathological features of the study population. The mean age of patients was 51.66±8.90 years. More cases were observed in the left breast (32 cases, 64%) compared to the right breast (18 cases, 36%). Among the 50 cases analyzed, 11 cases (22%) showed no residual disease. Residual disease was present in both the breast and lymph nodes in 22 cases (44%), in the breast alone in 15 cases (30%), and in the lymph nodes alone in two cases (4%). Of the 39 cases with residual tumors, the majority (22 cases, 56%) were categorized as RCB II, while 16 cases (41%) were RCB III, and only one case (3%) was RCB I. Stratification of residual tumors based on TIL and Ki-67 revealed that 20 cases (51%) had low TIL and high Ki-67, indicating a more proliferative phenotype with less immune activity. Tumors with high TIL and low Ki-67, suggestive of a less proliferative and potentially more immunogenic state, were seen in 12 cases (31%). Residual tumors with high TIL with high Ki-67 (four cases, 10%) and low TIL and low Ki-67 (three cases, 8%) were less common.

**Table 1:** Clinicopathological features of cases included in the study The data are represented as n (%). NACT: neoadjuvant chemotherapy; RCB: Residual Cancer Burden; TIL: tumor-infiltrating lymphocyte (immune activity)

Variables	Values (n=50)
Age	51.66±8.90
Laterality	
Left breast	32 (64%)
Right breast	18 (36%)
Residual disease post-NACT (n=50)	
No residual disease	11 (22%)
Residual disease in the breast and lymph node	22 (44%)
Residual disease in the breast	15 (30%)
Residual disease in the lymph node	2 (4%)
RCB in specimens with residual tumor (n=39)	
RCB I	1 (3%)
RCB II	22 (56%)
RCB III	16 (41%)
Immune activity and proliferative capacity of the residual tumor (n=39)	
TIL low, Ki-67 high	20 (51%)
TIL high, Ki-67 low	12 (31%)
TIL high, Ki-67 high	4 (10%)
TIL low, Ki-67 low	3 (8%)

Histological changes post-NACT

Table 2 highlights the stromal alterations observed in the post-NACT specimens.

**Table 2:** Stromal changes after chemotherapy in the breast tissue (n=50) The data are represented as n (%).

Stromal change	No. of cases
Fibrosis	31 (62%)
Hemosiderin-laden macrophages	14 (28%)
Foamy histiocyte	16 (32%)
Necrosis	19 (38%)
Angiogenesis	11 (22%)
Hyalinization of the wall of blood vessels	11 (22%)
Calcification	4 (8%)
Cholesterol clefts	12 (27%)

**Figure 1:**
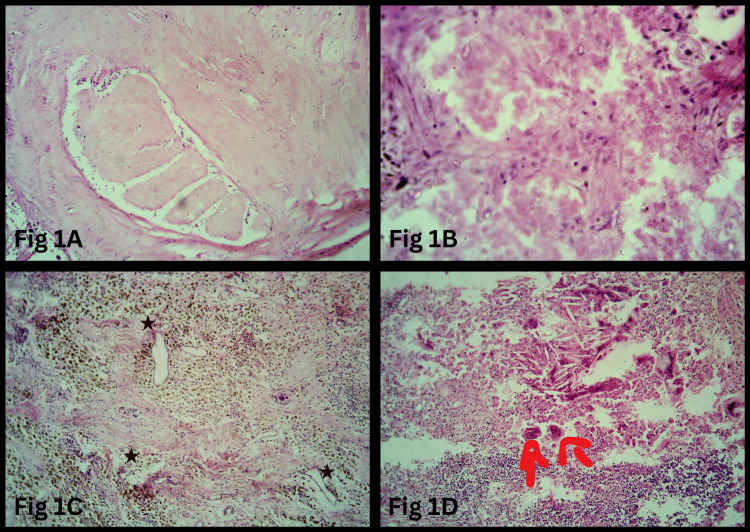
Histopathological changes in the tumor bed post-NACT (A) Dense stromal fibrosis within the tumor bed (H&E, 200×). (B) Tumor cell necrosis, indicating a response to chemotherapy (H&E, 200×). (C) Sheets of hemosiderin-laden macrophages and neoangiogenesis (H&E, 100×) (*). (D) Tumor regression changes characterized by cholesterol clefts, foamy macrophages, giant cells (red arrows), and lymphocytic infiltrates (H&E, 100×). NACT: neoadjuvant chemotherapy; H&E: hematoxylin and eosin

Fibrosis (Figure 1A) was the most common stromal change, observed in 31 cases (62%), followed by necrosis (Figure 1B) in 19 cases (38%) and infiltration by foamy histiocytes in 16 cases (32%). Tumor regression changes included infiltration by hemosiderin-laden macrophages (Figure 1C) in 14 cases (28%), cholesterol clefts (Figure 1D) in 12 cases (27%), angiogenesis in 11 cases (22%), hyalinization of blood vessel walls in 11 cases (22%), and calcification in four cases (8%).

Table 3 compares the cytological features observed in malignant (n=39) and benign (n=39) epithelial cells following chemotherapy in cases with residual tumor cells.

**Table 3:** Cytological changes in malignant and benign epithelial cells after chemotherapy in cases with residual tumor *Fisher's exact test was used to calculate p-value. The chi-squared test was used for other variables.

Features	Malignant (n=39)	Benign (n=39)	P-value
Foamy cytoplasm	16 (41%)	2 (5%)	0.00*
Cytoplasmic shrinkage	12 (31%)	22 (56%)	0.02
Increased nuclear-cytoplasmic ratio	14 (36%)	20 (51%)	0.17
Hyperchromatic nuclei	26 (67%)	6 (15%)	0.00
Prominent nucleoli	26 (67%)	4 (10%)	0.00*

**Figure 2:**
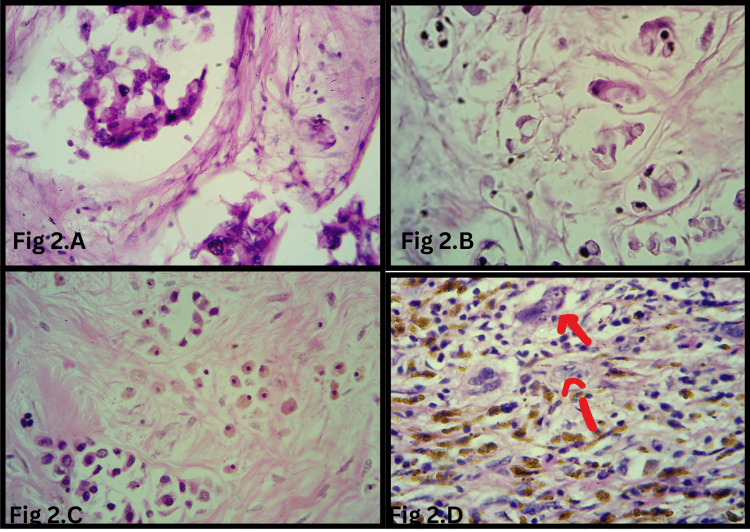
Cellular changes in the tumor bed post-NACT (A) Residual tumor cells with foamy cytoplasm and frayed edges (H&E, 400×). (B) Tumor cells displaying vacuolated cytoplasm (H&E, 400×). (C) Dyscohesive tumor cells with hyperchromatic nuclei (H&E, 400×). (D) Single scattered atypical cells exhibiting bizarre nuclei, irregular nuclear membranes, and prominent nucleoli (red arrows) (H&E, 400×). NACT: neoadjuvant chemotherapy; H&E: hematoxylin and eosin

Malignant epithelial cells demonstrated a significantly higher presence of foamy cytoplasm (16 cases (41%) vs. two cases (5%); p=0.0003) (Figure 2A, 2B), nuclear hyperchromasia (26 cases (67%) vs. six cases (15%); p=0.0001) (Figure 2C), and prominent nucleoli (26 cases (67%) vs. four cases (10%); p=0.0001) (Figure 2D) compared to benign epithelial cells. Conversely, cytoplasmic shrinkage was more frequently observed in benign cells than in malignant cells (22 cases (56%) vs. 12 cases (31%); p=0.02).

No statistically significant difference was observed between malignant and benign cells for an increased nuclear-cytoplasmic ratio (14 cases (36%) vs. 20 cases (51%); p=0.17). Among the lymph nodes analyzed, residual disease was present in 24 cases (58%). Necrosis was the most frequent histological change, observed in 34 cases (68%), followed by fibrosis in 28 cases (56%) and angiogenesis in 19 cases (38%). Less common findings included giant cells in four cases (8%) and granulomas in three cases (6%).

Immune activity and proliferative capacity

**Figure 3:**
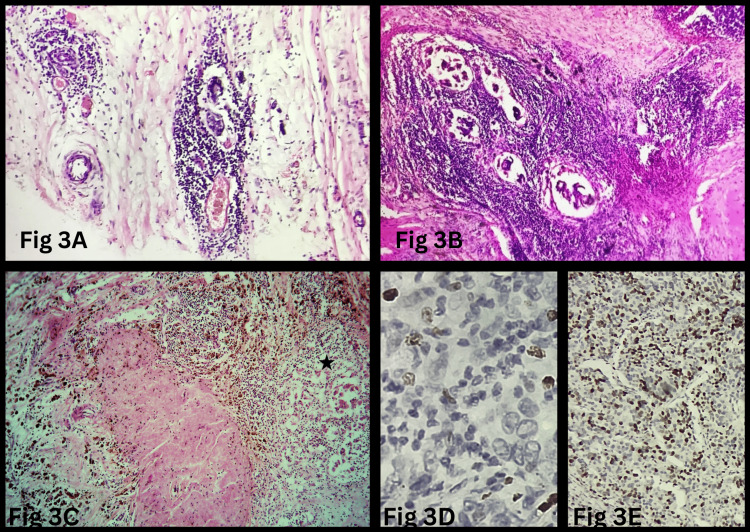
Tumor-infiltrating lymphocytes and proliferation index in residual tumor cells post-NACT (A, B) Dense tumor-infiltrating lymphocytes around the tumor nests (H&E, 200×). (C) Dense tumor-infiltrating lymphocytes around the tumor cells (*) with ongoing necrosis, with adjacent foci showing regression and fibrosis (H&E, 400×). (D) Residual tumor cells with low Ki-67 index (Ki-67 IHC, 400×). (E) Residual tumor cells with high Ki-67 index (Ki-67 IHC, 400×). NACT: neoadjuvant chemotherapy; H&E: hematoxylin and eosin; IHC: immunohistochemistry

Histopathological analysis of the residual tumor in 39 cases showed that 16 cases (41%) had high TIL, while 23 cases (59%) had low TIL. Ki-67 expression was high in 24 cases (61.5%), low in 14 cases (36%), and intermediate in one case (2.5%) (Figure 3).

Table 4 provides the association between stromal TILs, RCB, and the Ki-67 index. A significant inverse relationship was found between TIL levels and Ki-67 expression (p=0.0002), as tumors with low TIL were more likely to have high Ki-67 expression (20 cases, 51%), whereas those with high TIL more frequently exhibited low Ki-67 expression (12 cases, 31%). No significant correlation was observed between TIL levels and age (p=0.08), RCB (p=0.34), or lymph node involvement (p=0.98).

**Table 4:** Association between stromal TIL levels (low vs. high) and clinicopathological characteristics and Ki-67 index Data are presented as n (%). *Fisher's exact test was used to calculate p-value. The chi-squared test was used for other variables. For statistical analysis, one case with intermediate Ki-67 was taken together with low Ki-67(#). TIL: tumor-infiltrating lymphocyte

Characteristics	TIL low (n=23)	TIL high (n=16)	P-value
Age ≤52	15 (65%)	6 (38%)	0.08
Age >52	8 (35%)	10 (62%)	
RCB			
Low	15 (65%)	8 (50%)	0.34
High	8 (35%)	8 (50%)	
Lymph nodes			
Present	13 (57%)	9 (56%)	0.98
Absent	10 (43%)	7 (44%)	
Ki-67 index			
Low^#^	3 (13%)	12 (75%)	0.00*
High	20 (87%)	4 (25%)	

## Discussion

This study analyzed histopathological changes in the tumor bed post-NACT and quantified RCB, TIL levels, and the Ki-67 index in MRM specimens from 50 breast cancer patients following NACT. pCR was achieved in only 11 cases (22%), consistent with findings by Perubhotla et al. [14] and Sethi et al. [15]. A substantial RCB was observed (RCB II: 22 cases (56%); RCB III: 16 cases (41%)), reflecting challenges in achieving a complete pathological response, as noted in previous Indian studies [14]. These findings highlight the need for therapies targeting resistant tumor subpopulations and reinforce the potential of RCB as a valuable metric for assessing the need for additional treatment.

Histological evaluation of the tumor bed identified fibrosis as the most common stromal change in post-NACT specimens, aligning with previous studies [14,15]. Fibrosis may reflect a fibroinflammatory microenvironment that restricts tumor growth and promotes regression. It can also influence immune cell infiltration into the tumor bed and may represent the replacement of malignant cells following chemotherapy-induced cell death [16]. As a crucial component of cancer stroma research, fibrosis is known to play complex and sometimes opposing roles in tumor progression. Future studies are needed to determine whether fibrosis is a driver or a consequence of tumor regression, with significant implications for prognosis and therapeutic strategies. The high prevalence of necrosis in 19 cases (38%) likely reflects the cytotoxic effects of NACT. Angiogenesis (11 cases, 22%) may represent wound healing and tissue remodeling processes in response to treatment. Giant cells and granulomas suggest a chronic inflammatory response and potential involvement of immune-mediated mechanisms.

Cytopathological features, such as hyperchromasia, prominent nucleoli, karyorrhexis, and nuclear pleomorphism, were more significantly seen in malignant epithelial cells than in benign cells in breast specimens. These cytological changes were observed independently by many observers previously [14,17]. The acquisition of foamy cytoplasm by malignant cells may reflect altered metabolic activity or cellular stress induced by chemotherapy. The higher frequency of cytoplasmic shrinkage in benign cells could be attributed to various factors, including cellular senescence or different responses to treatment. The lack of significant differences in the nuclear-cytoplasmic ratio suggests these features may not be reliable indicators of malignancy in this context. Thus, the histopathological evaluation of residual disease in post-NACT specimens is challenging for pathologists. Reliance on immunohistochemistry is suggested for the definite identification of residual malignancy.

Survival and relapse rates of post-pCR vary across breast cancer subtypes [18], raising concerns about relying on pCR as a definitive surrogate endpoint. Some patients relapse despite achieving pCR, while others without pCR experience long-term survival. The accurate quantification and characterization of residual tumors are crucial for guiding further treatment. Chemotherapy-resistant tumor cells may harbor complex genetic abnormalities or therapy-induced genomic alterations that promote survival and proliferation. Additionally, the tumor microenvironment dictates tumor cell behavior.

In our study, 20 cases (51%) of the residual tumors were highly proliferative with low immune activity, explaining the poor response to chemotherapy. Ki-67 expression was significantly higher in TIL-low cases compared to TIL-high cases. Wang et al. observed that triple-negative breast cancers (TNBCs) that retained the high proliferation rate post-NACT with low TIL in RD were associated with worse OS and disease-free survival [19]. Higher posttreatment Ki-67 levels were found to be associated with a higher risk of relapse during the first three years after surgery in hormone receptor-positive cases [13]. These tumors may benefit from the addition of checkpoint inhibitors in addition to immunotherapy.

The prognostic value of TILs in residual cancer post-NACT remains uncertain due to conflicting evidence. In our study, 12 cases (31%) of tumors had high TILs and low Ki-67, indicating a favorable microenvironment with suppressed tumor proliferation. In contrast, four cases (10%) displayed both high TILs and high Ki-67, suggesting an active immune response coexisting with aggressive tumor growth. Dieci et al. [9] reported a significant correlation between increased TILs and improved metastasis-free survival and OS compared to patients with low TILs. In contrast, Hamy et al. [20] observed the opposite in a large cohort of HER2+ patients, where high post-NACT TIL levels were linked to worse prognoses. Immune escape by the downregulation of major histocompatibility complex (MHC) and immune editing may be the potential mechanisms that can explain the persistence of tumor cells despite a brisk immune response. Post-NACT TILs may be functionally impaired due to an immunosuppressive tumor microenvironment, which includes inhibitory checkpoints, immunosuppressive cells, and cytokines. The interaction of tumor cells with immune checkpoint inhibitors like PDL-1 may potentially enable tumors to evade immune surveillance in tissues [21]. In three cases (8%), tumors had low immune activity and reduced proliferative capacity. Tumors with low Ki-67 indicate slower-growing tumors with potentially less sensitivity to chemotherapy [22]. Reduced TIL levels in these cases may be due to the downregulation of the immune system due to reduced damage-associated molecular patterns (DAMPs) because of reduced tumor cell killing by chemotherapy. Reduced TIL may also be due to direct suppression by chemotherapy [23]. Thus, evaluating Ki-67 and TIL in residual tumors, along with tumor response and immune changes induced by NACT, can stratify residual disease into distinct subgroups and provide insights into novel therapeutic interventions.

While molecular subtypes and specific NACT regimens are critical factors in breast cancer biology and treatment response, our study design intentionally focuses on broader histological and immunological changes in the tumor bed post-NACT. This approach allows us to capture universal patterns of tumor proliferative capacity (Ki-67) and immune activity (TILs) that transcend molecular subtypes and NACT regimen variability. This foundational perspective is essential for unraveling the complex interplay between residual tumor cells, chemotherapy effects, and the immune microenvironment, paving the way for future studies that integrate molecular subtyping and regimen-specific analyses to refine personalized treatment strategies.

The study highlights the importance of integrating pathological response assessments (RCB index) with immune and proliferative markers in understanding the treatment resistance for risk stratification and personalized treatment approaches in post-NACT cases. The adoption of standardized protocols enhances the reliability of our results.

Limitations

The limitation of our study is the small sample size and unicentric design, which affects the generalizability of the findings. Lack of correlation with pretreatment, histological, and immunohistochemical parameters that are known to be modified post-NACT is also a major limitation. The lack of correlation of findings with NACT regimens, molecular subtypes, and the absence of follow-up data limits the ability to assess the true prognostic impact of histological changes and immune markers.

## Conclusions

NACT strategies have achieved significantly high pCR rates, yet they have not uniformly translated into improved survival outcomes. A comprehensive post-NACT assessment incorporating RCB, Ki-67, and TIL offers a valuable approach to understanding residual tumor biology. This morphologic evaluation is feasible in routine laboratory settings and enables patient stratification into distinct subgroups, facilitating decisions on additional personalized treatment decisions post-NACT that are neither excessive nor inadequate. Our study establishes a foundation for future research aimed at refining prognostic and predictive models for breast cancer post-NACT. While our study has translational relevance, its conclusions must be validated through large-scale studies, long-term patient follow-up, and dedicated clinical trials to ensure broader applicability and clinical impact.
